# Comparison of all-cause mortality associated with non-alcoholic fatty liver disease and metabolic dysfunction-associated fatty liver disease in Taiwan MJ cohort

**DOI:** 10.4178/epih.e2024024

**Published:** 2024-01-21

**Authors:** Wei-Chun Cheng, Hua-Fen Chen, Hsiu-Chi Cheng, Chung-Yi Li

**Affiliations:** 1 Department of Internal Medicine, National Cheng Kung University College of Medicine, Tainan, Taiwan; 2 Department of Public Health, National Cheng Kung University College of Medicine, Tainan, Taiwan; 3 Department of Gastroenterology and Hepatology, Tainan Hospital, Ministry of Health and Welfare, Tainan, Taiwan; 4 Department of Endocrinology, Far Eastern Memorial Hospital, New Taipei City, Taiwan; 5 School of Medicine and Department of Public Health, Fujen Catholic University College of Medicine, New Taipei City, Taiwan; 6 Institute of Clinical Medicine, National Cheng Kung University College of Medicine, Tainan, Taiwan; 7 Institute of Molecular Medicine, National Cheng Kung University College of Medicine, Tainan, Taiwan; 8 Department of Public Health, China Medical University College of Public Health, Taichung, Taiwan; 9 Department of Healthcare Administration, Asia University College of Medical and Health Science, Taichung, Taiwan

**Keywords:** Non-alcoholic fatty liver disease, Metabolic dysfunction-associated fatty liver disease, Hepatic fibrosis, Mortality

## Abstract

**OBJECTIVES:**

The global burden of non-alcoholic fatty liver disease (NAFLD) is rising. An alternative term, metabolic dysfunction-associated fatty liver disease (MAFLD), instead highlights the associated metabolic risks. This cohort study examined patient classifications under NAFLD and MAFLD criteria and their associations with all-cause mortality.

**METHODS:**

Participants who attended a paid health check-up (2012-2015) were included. Hepatic steatosis (HS) was diagnosed ultrasonographically. NAFLD was defined as HS without secondary causes, while MAFLD involved HS with overweight/obesity, type 2 diabetes mellitus, or ≥2 metabolic dysfunctions. Mortality was tracked via the Taiwan Death Registry until November 30, 2022.

**RESULTS:**

Of 118,915 participants, 36.9% had NAFLD, 40.2% had MAFLD, and 32.9% met both definitions. Participants with NAFLD alone had lower mortality, and those with MAFLD alone had higher mortality, than individuals with both conditions. After adjustment for potential confounders, the hazard ratios (HRs) for all-cause mortality were 1.08 (95% confidence interval [CI], 0.78 to 1.48) for NAFLD alone and 1.26 (95% CI, 1.09 to 1.47) for MAFLD alone, relative to both conditions. Advanced fibrosis conferred greater mortality risk, with HRs of 1.93 (95% CI, 1.44 to 2.58) and 2.08 (95% CI, 1.61 to 2.70) for advanced fibrotic NAFLD and MAFLD, respectively. Key mortality risk factors for NAFLD and MAFLD included older age, unmarried status, higher body mass index, smoking, diabetes mellitus, chronic kidney disease, and advanced fibrosis.

**CONCLUSIONS:**

All-cause mortality in NAFLD and/or MAFLD was linked to cardiometabolic covariates, with risk attenuated after multivariable adjustment. A high fibrosis-4 index score, indicating fibrosis, could identify fatty liver disease cases involving elevated mortality risk.

## GRAPHICAL ABSTRACT

[Fig f5-epih-46-e2024024]

## Key Message

In a cohort study involving 118,915 Taiwanese participants in a paid health check-up program, approximately one-third met both NAFLD (non-alcoholic fatty liver disease) and MAFLD (metabolic dysfunction-associated fatty liver disease) criteria. All-cause mortality associated with NAFLD and/or MAFLD correlated with cardiometabolic factors, though the risk attenuated following multivariable adjustment. A high fibrosis-4 index score, suggestive of liver fibrosis, was predictive of increased mortality risk in FLD (fatty liver disease) cases.

## INTRODUCTION

The global burden of metabolic dysfunction and its associated disorders has risen over recent years [1]. Fatty liver diseases (FLDs), particularly non-alcoholic fatty liver disease (NAFLD), are the predominant hepatic manifestations of these disorders [2]. NAFLD is characterized by the accumulation of fat and damage to hepatocytes and is associated with excessive calorie intake and reduced energy expenditure [3]. NAFLD is marked by fat accumulation in the liver, not attributable to excessive alcohol consumption or other underlying medical conditions. It is closely associated with metabolic dysfunction, including insulin resistance, hypertension, hyperlipidemia, metabolic syndrome, and type 2 diabetes [4]. However, for a disease affecting 20% to 25% of the global adult population and with serious potential health consequences [5], definition via negative criteria seems counterintuitive. Moreover, the diagnostic criterion that excludes alcohol use is highly culture-dependent [6]. Additionally, ruling out other liver comorbidities such as viral hepatitis, drug-induced liver injury, or autoimmune processes may not be straightforward during routine outpatient visits.

The newly proposed definition of metabolic dysfunction-associated fatty liver disease (MAFLD) adopts a positive diagnostic approach by including individuals with hepatic steatosis who are also overweight or obese, have diabetes, or exhibit metabolic dysfunctions [3]. The associations of clinical outcomes with both NAFLD and MAFLD warrant further exploration in real-world studies [7-9]. Previous research has shown that the incidence of cardiovascular disease increases when metabolic risk factors are incorporated into the disease definition [7,9]. However, the influence of disease progression on mortality outcomes in variously defined FLDs has not been sufficiently explored [8], particularly in Asian populations.

This study was conducted to compare the characteristics of patients with FLDs based on the definitions of NAFLD and MAFLD. Additionally, we sought to investigate the mortality outcomes, with a focus on all-cause mortality, associated with NAFLD and/or MAFLD. Participants were divided into 4 groups: those with both FLDs, those with NAFLD alone, those with MAFLD alone, and those without FLDs, within a Taiwanese health check-up cohort.

## MATERIALS AND METHODS

### Study design and sample

This study was a retrospective cohort analysis utilizing a large cohort of health check-up participants in Taiwan, specifically the MJ cohort. Participants were prospectively enrolled, with a detailed record of demographic information, lifestyle factors, and medical history collected through self-administered questionnaires [10]. Furthermore, a linkage was established between the MJ cohort and the Taiwan Death Registry (TDR). In Taiwan, it is a legal requirement to register all deaths within 10 days, which ensures a high level of completeness for the TDR. Leveraging these 2 datasets, we were able to explore long-term mortality in relation to NAFLD and/or MAFLD, while also considering lifestyle factors closely associated with metabolic dysfunction disorders, comorbidities, FLDs, and mortality [11-14].

**Figure 1.**
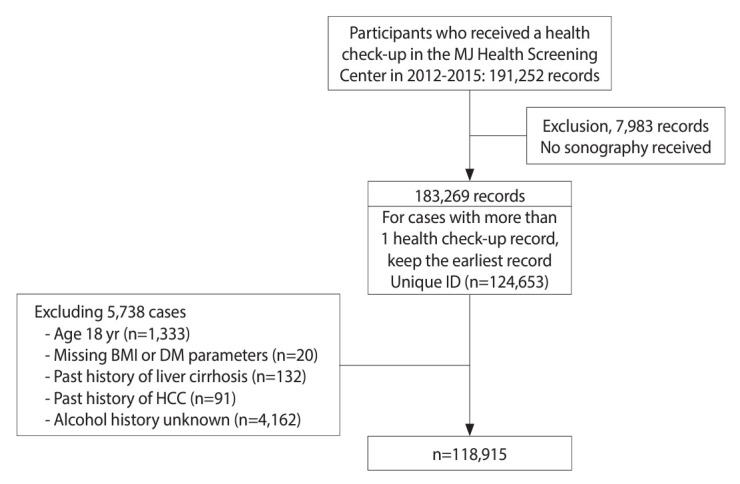
Flow diagram of participant enrollment for this study. BMI, body mass index; DM, diabetes mellitus; HCC, hepatocellular carcinoma.

We retrieved records from MJ clinics for 191,252 individuals who attended health check-ups from 2012 to 2015. We excluded 7,983 records that lacked ultrasonography data and utilized the earliest record (i.e., the index health check-up) for those who underwent more than 1 check-up during the study years. This resulted in 124,653 study participants (records) included in this study. Additional exclusion criteria were as follows: individuals under 18 years of age (n= 1,333), missing information on body mass index (BMI) or diabetes-related biochemical parameters (n= 20), reported history of liver cirrhosis (n= 132) or hepatocellular carcinoma (n= 91), and unknown alcohol intake history (n= 4,162). After applying these criteria, a total of 118,915 records were analyzed. The flowchart depicting the enrollment of study participants is shown in Figure 1.

### Definitions of non-alcoholic fatty liver disease and metabolic dysfunction-associated fatty liver disease

Hepatic steatosis was diagnosed using abdominal ultrasonography performed by well-trained clinicians at MJ clinics, consistent with the methods reported in previous studies [13,15]. Despite abdominal ultrasonography being susceptible to inter-rater reliability issues and occasional technical difficulties in interpretation, it remains the preferred method for most large-scale epidemiological studies where gold standards such as magnetic resonance imaging or liver biopsy are impractical [6,16].

NAFLD was defined as hepatic steatosis identified through ultrasonography, in the absence of secondary causes such as excessive alcohol consumption, viral hepatitis, medications known to cause steatosis, or other concurrent liver diseases. Alcohol consumption status was categorized as never, former, or current drinking. Individuals who currently consumed 2 or more alcoholic beverages per day, on at least 3 days per week, and had done so for over 1 year [11]—corresponding to a weekly alcohol intake of more than 70 g to 140 g [6]—were considered to have excessive alcohol intake. Consequently, they were excluded from the NAFLD group.

Based on international expert consensus [3], MAFLD was defined as cases with hepatic steatosis in individuals who had a BMI of at least 23 kg/m^2^ (indicating overweight or obesity), diagnosed type 2 diabetes mellitus (T2DM), or 2 or more metabolic dysfunctions. T2DM was defined as having fasting glucose levels of at least 126 mg/dL, serum hemoglobin A1c of at least 6.5%, or a self-reported previous diagnosis. Metabolic dysfunctions included: (1) waist circumference of 90 cm or more for male and 80 cm or more for female; (2) blood pressure of 130/85 mmHg or higher, or treatment with specific drugs; (3) plasma triglycerides of 150 mg/dL or higher, or treatment with specific drugs; (4) plasma high-density lipoprotein cholesterol levels below 40 mg/dL for male and below 50 mg/dL for female, or treatment with specific drugs; (5) prediabetes, characterized by fasting glucose levels of 100 mg/dL to 125 mg/dL or hemoglobin A1c levels of 5.7% to 6.4%; (6) homeostatic model assessment for insulin resistance score of 2.5 or higher; and (7) plasma high-sensitivity C-reactive protein levels above 2 mg/L.

By combining the definitions of NAFLD and MAFLD, the study participants were categorized into 4 groups. Those who met neither the NAFLD nor MAFLD criteria were classified as “non-FLD”, while individuals who met both sets of criteria were classified as having “both FLDs”. Participants categorized as “NAFLD only” were those who fulfilled the criteria for NAFLD but did not have overweight/obesity or T2DM, and who had fewer than 2 of the metabolic risk factors previously mentioned. Similarly, the “MAFLD only” group consisted of participants who met the criteria for MAFLD but had other causes of hepatic steatosis, such as excessive alcohol intake, drug-induced liver injury, or viral hepatitis.

### Covariates

Socio-demographic characteristics and lifestyle information were collected through self-administered questionnaires completed on the day of the health check-up. Like alcohol intake, smoking status was categorized as never, former, or current. Regular exercise was defined as engaging in at least 30 minutes of physical activity once every 2-3 days or more. Anthropometric measurements (including body weight, height, and waist circumference), as well as blood tests, were performed according to the standard protocols of the MJ Health Management Institution. To estimate the glomerular filtration rate, we employed the modified Modification of Diet in Renal Disease Study equation: 175 × Cr^-1.154^× Age^-0.203^× (0.742, if female)× (1.212, if Black), where Cr represents the serum creatinine level [17]. Individuals with an estimated glomerular filtration rate of less than 60 mL/min/1.73 m^2^ were identified as having chronic kidney disease. Chronic hepatitis B was defined by a positive hepatitis B surface antigen test or a self-reported history of hepatitis B. Chronic hepatitis C was identified by a positive anti-hepatitis C virus antibody test or a reported history of hepatitis C. Fibrosis-4 index (Fib-4) was calculated using the formula: age (years× AST [U/L]/(platelets [10^9^/L]× (ALT [U/L])^1/2^, to assess liver fibrosis status [18]. A cut-off value of 2.67 was used to define advanced fibrosis [19].

### Follow-up and mortality

Mortality status was confirmed by linking each participant’s personal identification number to the TDR. The follow-up period was calculated in months from the date of the health check-up (the index date) to the date of death (if applicable) or to the last day of the study period (November 30, 2022), whichever occurred first. The underlying cause of death was recorded in the TDR according to International Classification of Diseases, 10th revision (ICD-10) codes. These were categorized as cancer-related mortality (ICD-10: C00-C97), cardiovascular disease-related mortality (ICD-10: I01-I02.0, I05-I09, I20-I25, I27, I30-I52, I60-I69), trauma and self-harm mortality (ICD-10: V01-X59, X60-X84, Y85-Y86, Y87.0), and liver disease-related mortality (ICD10: K70, K73-K74).

### Statistical analysis

For descriptive statistics comparing characteristics across FLD classifications, we used means± standard deviations to represent continuous variables and numbers and percentages for categorical variables. Mortality rates were calculated as the number of events per 10,000 person-years (PY) of follow-up, assuming a Poisson distribution. We estimated cumulative all-cause mortality rates using the Kaplan-Meier method and compared these rates among FLD groups with log-rank tests. To estimate hazard ratios (HRs) and 95% confidence intervals (CIs) for all-cause mortality associated with various FLD classifications, Cox proportional hazards models were employed. We constructed multiple Cox regression models in a sequential manner to adjust for age, sex, socioeconomic factors, BMI, health behaviors, and comorbidities. Since some subcategories of diabetes mellitus and viral hepatitis status included no participants, we did not adjust for these variables in the models. The proportional hazards assumption was supported by plots of log(-log(survival function)) versus log(time) for the primary comparisons in the study, specifically “NAFLD only” versus “both FLDs” and “MAFLD only” versus “both FLDs.” The absence of multicollinearity was confirmed in the fully adjusted model by checking the variance inflation factor and condition index. Data analyses were performed using SAS version 9.4 (SAS Institute Inc., Cary, NC, USA) and R version 4.2.2 (R Foundation for Statistical Computing, Vienna, Austria). A significance level of 0.05 was used for all statistical tests.

### Ethics statement

This study received approval from the Institutional Review Board of National Cheng Kung University Hospital, Tainan, Taiwan (approval No. B-ER-110-456), with a waiver of informed consent.

## RESULTS

**Figure 2.**
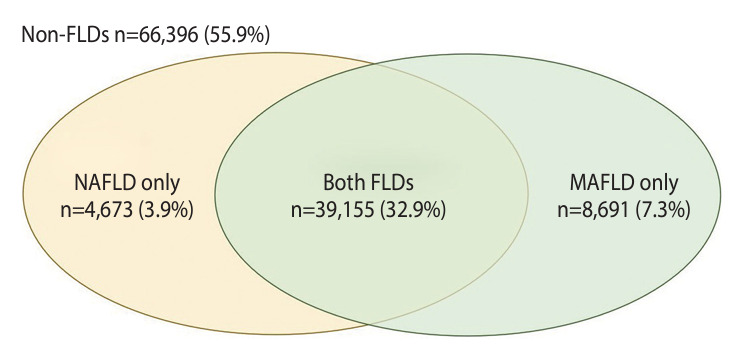
Prevalence and distribution of study participants according to NAFLD and MAFLD status. FLD, fatty liver disease; MAFLD, metabolic dysfunction-associated fatty liver disease; NAFLD, nonalcoholic fatty liver disease.

Based on ultrasonography and reviews of medical history, 43,828 participants (36.9%) were identified as having NAFLD, as shown in Supplementary Material 1. In contrast, 47,846 participants (40.2%) met the criteria for MAFLD, depicted in Supplementary Material 2. By applying both NAFLD and MAFLD criteria (Supplementary Material 3), participants were further categorized into 4 groups. Figure 2 illustrates the numbers and proportions of these groups, illustrating the prevalence of the FLDs. Of the study sample, 66,396 cases (55.9%) did not meet the criteria for either NAFLD or MAFLD and were thus classified as “non-FLD”. Conversely, 39,155 participants (32.9%) met the criteria for both FLDs. The remaining participants included those with NAFLD only, numbering 4,673 (3.9%), and those with MAFLD only, totaling 8,691 (7.3%).

Table 1 compares the socio-demographic characteristics and clinical parameters of the 4 study groups. Participants with FLDs were generally older and more likely to be male compared to those without FLDs, with the highest average age and greatest proportion of male participants observed in the group with MAFLD only. Marital and educational status also varied across the study groups, with non-FLD participants more often being unmarried. Higher educational attainment was noted among those without FLD or with NAFLD only. The non-FLD participants also displayed a lower prevalence of current or former smokers, diabetes, and prediabetes compared to the other 3 groups. Based on its negative diagnostic criteria, NAFLD cases excluded individuals with meaningful alcohol intake or chronic viral hepatitis. Conversely, those with steatosis accompanied by overweight/obesity or diabetes were included in the MAFLD groups (i.e., “MAFLD only” and “both FLDs”). Therefore, all cases in the “NAFLD only” group had no meaningful alcohol intake, overweight/obesity, diabetes, or viral hepatitis. In contrast, individuals with fatty liver along with substantial alcohol intake or viral hepatitis were categorized as “MAFLD only.” Regarding fibrosis status, as assessed by Fib-4, the “MAFLD only” group had the highest proportion of advanced fibrosis (Fib-4≥ 2.67), compared to the “both FLDs” and “NAFLD only” groups.

**Figure 3.**
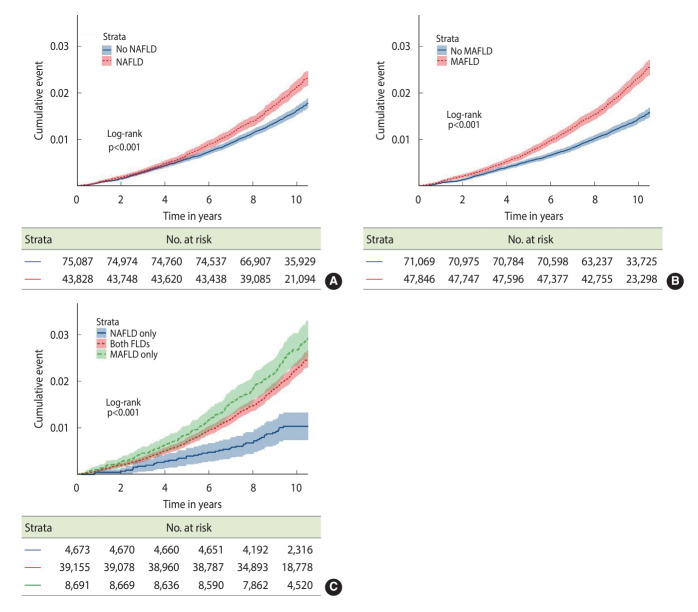
Comparisons of cumulative all-cause mortality rates among participants (A) with or without NAFLD; (B) with or without MAFLD; (C) with fatty liver disease (FLD), categorized by NAFLD and MAFLD classification. MAFLD, metabolic dysfunction-associated fatty liver disease; NAFLD, non-alcoholic fatty liver disease.

After a mean follow-up period of 9.6± 1.2 years, 2,037 participants experienced all-cause mortality, corresponding to a mortality rate of 17.8 per 10,000 PY. The highest and lowest mortality rates were observed in participants with MAFLD only (27.0 per 10,000 PY) and NAFLD only (10.0 per 10,000 PY), respectively (Table 2). Among all study participants, the 3 leading causes of death were malignancies (8.0 per 10,000 PY), cardiovascular disease (2.8 per 10,000 PY), and trauma and self-harm (1.5 per 10,000 PY). The mortality rate due to chronic liver disease or cirrhosis was 0.2 per 10,000 PY (Supplementary Material 4 for details of cause-specific mortality). Figure 3 displays the Kaplan-Meier survival curves for all-cause mortality across various study groups. Compared to those without NAFLD, participants with NAFLD exhibited a significantly higher cumulative mortality rate during the follow-up period (Figure 3A). Similarly, participants with MAFLD also demonstrated a significantly higher cumulative mortality rate relative to those without MAFLD (Figure 3B).

Table 2 presents the mortality rates associated with NAFLD/MAFLD and Fib-4 status. Individuals without NAFLD had an all-cause mortality rate of 16.1 per 10,000 PY, which was lower than the rate for those with NAFLD, at 20.9 per 10,000 PY. The all-cause mortality rate rose in conjunction with increasing Fib-4 value. Participants with the lowest fibrosis score (Fib-4< 1.30) had a mortality rate of 12.4 per 10,000 PY, while the group with the highest fibrosis score (Fib-4≥2.67) experienced a rate of 270.0 per 10,000 PY. The all-cause mortality rate for individuals without MAFLD was 14.3 per 10,000 PY. In contrast, participants with MAFLD had mortality rates of 13.5, 64.5, and 231.0 per 10,000 PY for Fib-4 scores of < 1.30, 1.30 to 2.67, and ≥ 2.67, respectively.

We additionally examined the mortality differences among FLD cases defined by the differing criteria for NAFLD and MAFLD. Figure 3C illustrates that, compared to cases meeting both FLD definitions, those with only NAFLD had a lower cumulative mortality rate, with a significantly reduced HR of 0.45 (95% CI, 0.33 to 0.61) (Table 3). Patients with MAFLD alone had a higher cumulative mortality rate, with an HR of 1.21 (95% CI, 1.04 to 1.40). After adjusting for all covariates, the HR for NAFLD alone was no longer statistically significant, whereas the increased risk for MAFLD alone persisted, with an HR of 1.26 (95% CI, 1.09 to 1.47). Table 3 and Supplementary Material 5 further reveal that fibrosis status was significantly associated with long-term mortality. The mortality hazard increased in a dose-response relationship with advancing fibrosis, regardless of whether NAFLD or MAFLD definitions were applied, compared to the subgroup with the least fibrosis. This effect remained significant even after adjusting for potential confounders. FLD participants with NAFLD and Fib-4≥ 2.67 had a significantly elevated covariate-adjusted HR of 1.93 (95% CI, 1.44 to 2.58) relative to participants with low fibrosis. For participants with MAFLD and Fib-4≥ 2.67, the HR was 2.08 (95% CI, 1.61 to 2.70).

**Figure 4.**
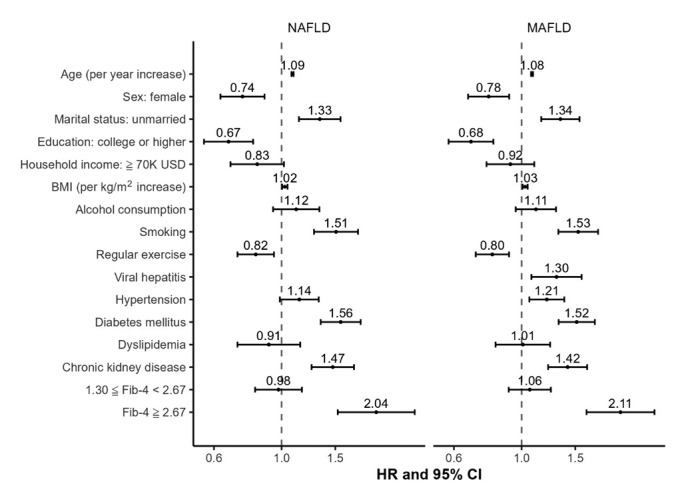
Comparisons of multivariable-adjusted hazard ratios for all-cause mortality among participants with NAFLD and MAFLD. USD, US dollar; BMI, body mass index; Fib-4, fibrosis-4 score; HR, hazard ratio; CI, confidence interval; MAFLD, metabolic dysfunction-associated fatty liver disease; NAFLD, non-alcoholic fatty liver disease.

Figure 4 separately examines the risk factors for all-cause mortality in participants with NAFLD and MAFLD. In both conditions, advanced age, unmarried status, higher BMI, smoking, diabetes mellitus, chronic kidney disease, and advanced fibrosis were identified as key risk factors for mortality. In contrast, female sex, higher education level, and regular exercise were found to be protective factors against mortality for both types of FLD. Notably, advanced age, smoking, diabetes mellitus, and advanced fibrosis were common risk factors for both cancer-related and cardiovascular disease-related mortality in patients with either form of FLD (Supplementary Material 6).

## DISCUSSION

This cohort study revealed that 36.9% of participants had NAFLD, 40.2% had MAFLD, and 32.9% met the criteria for both conditions. Individuals with NAFLD or MAFLD exhibited similar mortality HRs, compared to their non-FLD counterparts. Notably, participants with NAFLD alone exhibited lower mortality than those with both types of FLD, while those with only MAFLD demonstrated higher mortality. However, after adjusting for potential confounders, the reduced mortality HRs associated with NAFLD alone were no longer significant. For those with advanced fibrosis (Fib-4≥ 2.67), the adjusted mortality HRs were significantly higher at 1.93 (95% CI, 1.44 to 2.58) for advanced fibrotic NAFLD and 2.08 (95% CI, 1.61 to 2.70) for advanced fibrotic MAFLD, compared to the subgroups exhibiting less severe fibrosis. Key mortality risk factors for both NAFLD and MAFLD included older age, unmarried status, higher BMI, smoking, diabetes, chronic kidney disease, and advanced fibrosis.

Like previous studies [8,9,20], our results revealed that among individuals with FLD, those with NAFLD alone experienced lower mortality rates from all causes, cancer, and cardiovascular disease. To meet the criteria for NAFLD, individuals with substantial alcohol consumption or other liver diseases must be excluded; similarly, non-MAFLD cases must not exhibit overweight/obesity, diabetes, or most metabolic risk factors [3]. Our study demonstrated that by excluding these risk factors, the participants with NAFLD alone exhibited the lowest risks of all-cause mortality, cancer-related mortality, and cardiovascular disease-related mortality among those with FLD. In contrast, those with only MAFLD, which is excluded from the NAFLD definition due to substantial alcohol consumption or other liver disease and requires the presence of overweight/obesity, diabetes, or metabolic risk factors, showed the highest mortality risk. Notably, the HR reduction in the “NAFLD only” compared to the “both FLDs” group was not significant after adjusting for age, sex, metabolic abnormalities, and comorbidities. This underscores the impact of these factors on mortality outcomes in patients with NAFLD, both with and without MAFLD. This finding is consistent with previous research by Allen et al. [21], which highlighted the influence of highly dysmetabolic conditions on mortality risk in patients with NAFLD.

The study participants, who paid for health check-ups at MJ clinics, generally exhibited relatively high socioeconomic status. They were likely to possess comparatively high health literacy and awareness, which could lead to increased vigilance regarding minor health issues. This includes conditions such as fatty liver, detected via sonography, or metabolic dysfunctions. Prior research has shown that individuals who attend health check-ups tend to seek more outpatient care and have higher prescription rates for conditions like hypertension and dyslipidemia. This behavior is associated with a lower risk of cardiovascular disease [22]. Additionally, higher health literacy, greater self-efficacy in managing diseases, and positive health behaviors are interconnected and may contribute to reduced mortality risk [23,24]. Participants who pay for health check-ups and are aware of their medical conditions, such as metabolic dysfunction or FLD, might be relatively proactive in managing their health. This could involve lifestyle changes and controlling risk behaviors, such as decreasing alcohol intake, quitting smoking, and avoiding a sedentary lifestyle. Such a proactive stance could account for the observation that participants with NAFLD alone—those without alcohol use, other causes of hepatic steatosis, or metabolic risk factors—exhibited lower mortality rates compared to individuals without FLD.

The long-term prognosis of NAFLD and/or MAFLD has been investigated in several previous studies. Nguyen et al. [8] utilized the National Health and Nutrition Examination Survey III dataset, employing ultrasonography to define fatty liver status. They observed a trend of decreasing mortality from patients with MAFLD alone to those with both FLDs, and finally to those with only NAFLD. In our study, individuals with MAFLD alone displayed a viral hepatitis prevalence of over 58.0%, while only 18.6% reported excessive alcohol intake; even former and current alcohol users combined represented only 33.9%. In contrast, the United States study reported a 68.9% prevalence of substantial alcohol intake in the same category. Despite these differences, an elevated mortality risk was similarly observed in both investigations. Varying proportions of alcohol consumption and viral hepatitis may account for the differences in fibrosis prevalence between MAFLD groups in these 2 articles. MAFLD-associated cardiovascular disease outcomes have been reported in most previous studies [7,9,25]. Cardiovascular disease and malignancies, rather than liver-related events, have been shown to be the primary causes of death in patients with FLDs [25-27]. These earlier findings align with our observations in the present study.

Our findings align with those of Lee et al. [9], showing a similar pattern of male predominance in both NAFLD and MAFLD, with increased prevalence with age. Although our study population displayed a lower mean age compared to most previous reports [7-9], we observed that FLDs, particularly MAFLD, can serve as markers to identify high-risk groups for all-cause mortality. To our knowledge, our research represents the largest cohort study to use sonography-defined hepatic steatosis for characterizing the risk of mortality outcomes. While the increased risk of mortality associated with FLDs was less pronounced after adjusting for covariates, NAFLD and MAFLD retain utility as markers for identifying high-risk individuals. From a public health perspective, the identification of such markers is the most crucial step in secondary prevention [28]. Our study also compared risk factors for all-cause mortality in cases of NAFLD and MAFLD, revealing similar risk factors for mortality regardless of the definition employed. This indicates that, despite changes in definitions, risk reduction strategies should focus on consistent factors. This conclusion is further supported by the findings of Younossi et al. [25].

Our study suggests that Fib-4, as a marker of fibrosis, may serve as an indicator of future mortality risk in individuals with NAFLD or MAFLD. This finding is consistent with previous research. In cases of biopsy-proven NAFLD and/or non-alcoholic steatohepatitis, advanced fibrosis has been linked to an increased risk of all-cause mortality [29-31]. Our current study also indicates that advanced fibrosis, as defined by a Fib-4 score of 2.67 or higher, may be a risk marker for all-cause, cancer-related, and cardiovascular disease-related mortality in participants with MAFLD.

The present study had several strengths, including a robust cohort design with a large sample size. Additionally, hepatic steatosis in FLDs was determined through sonography, a practical method for epidemiological research. However, some limitations should be acknowledged. First, FLD status was evaluated solely at baseline, thus not accounting for the dynamic nature of metabolic dysfunction and hepatic steatosis. As mentioned previously, the health check-up participants in the study may also have possessed heightened health awareness. Altered lifestyles and health behaviors after the medical appointment could have led to exposure misclassification and underestimation of associations with all-cause mortality. Second, Fib-4 score was used as the indicator of hepatic fibrosis, with cut-off values that were primarily established for NAFLD rather than MAFLD [32]. Nonetheless, our study demonstrated a clear dose-response relationship between fibrosis status and mortality risk in MAFLD cases. Third, the limited number of cause-specific deaths restricted our analysis of causespecific mortality, except for mortality from cancer and cardiovascular disease. Fourth, the study was based on a self-paid health check-up cohort, potentially limiting the generalizability of these findings. As evident from our data, the study participants were relatively young, well-educated (more than 80% had a high school degree or above), and higher-income (with around 60% earning an annual income exceeding US$28,000, equivalent to Taiwan’s gross domestic product per capita in 2020). Finally, we included data from all health check-up participants during the study period, which largely minimized the potential for non-response bias. In addition, the classification of NAFLD and MAFLD was based on both laboratory work and imaging data; mortality data were retrieved from the TDR, which also provided reassurance of a low risk of exposure and outcome misclassification. Nonetheless, we were unable to consider a comprehensive list of potential confounders in the analysis, which could have introduced potential confounding to some extent. Additional research is required to validate our findings in this health check-up population.

In conclusion, this cohort study revealed that MAFLD (with or without concurrent NAFLD) was significantly linked to an increased risk of all-cause mortality among individuals with FLD. Key risk factors for all-cause mortality in both types of FLD included older age, unmarried status, high BMI, smoking, diabetes mellitus, chronic kidney disease, and advanced fibrosis, as indicated by Fib-4 score. This score, as a measure of fibrosis status, may be a valuable tool for identifying patients with FLDs who are at a high risk of mortality.

## Figures and Tables

**Figure f5-epih-46-e2024024:**
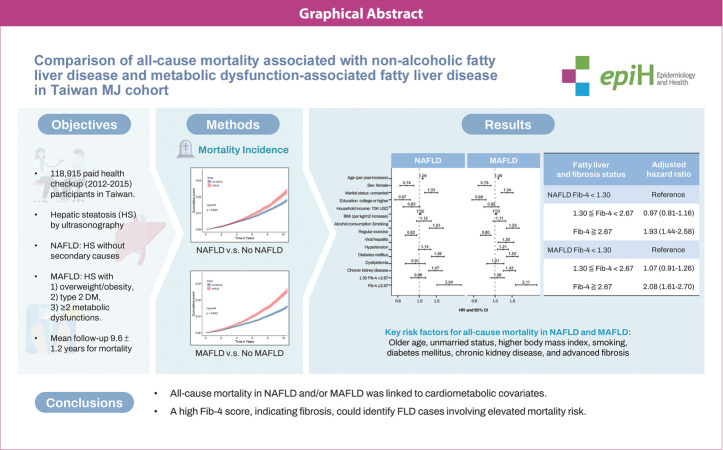


**Table 1.** Demographic variables and clinical parameters by fatty liver classification

Variables	Non-FLDs (n=66,396)	NAFLD only (n=4,673)	MAFLD only (n=8,691)	Both FLDs (n=39,155)
Age (yr)	39.5±12.1	41.5±10.6	46.0±10.8	45.3±12.6
Sex				
Male	24,791 (37.3)	2,046 (43.8)	6,633 (76.3)	25,532 (65.2)
Marital status				
Unmarried	26,325 (39.7)	1,500 (32.1)	1,730 (19.9)	10,276 (26.2)
Married	36,473 (54.9)	2,940 (62.9)	6,462 (74.4)	26,614 (68.0)
Unreported	3,598 (5.4)	233 (5.0)	499 (5.7)	2,265 (5.8)
Education				
Middle school or below	4,891 (7.3)	259 (5.6)	1,174 (13.5)	4,990 (13.9)
High school	22,536 (33.9)	1,657 (35.5)	3,622 (41.7)	14,208 (36.3)
College or above	37,783 (57.0)	2,674 (57.1)	3,728 (42.9)	18.665 (47.6)
Unreported	1,186 (1.8)	83 (1.8)	167 (1.9)	846 (2.2)
Annual household income (US dollar)				
<28,000	27,387 (41.3)	1,674 (35.8)	3,154 (36.3)	14,975 (38.3)
28,000-70,000	27,886 (41.9)	2,186 (46.8)	3,886 (44.7)	16,987 (43.4)
≥70,000	5,111 (7.7)	463 (9.9)	973 (11.2)	3,653 (9.3)
Unreported	6,012 (9.1)	350 (7.5)	678 (7.8)	3,540 (9.0)
Smoking				
Never	53,641 (80.8)	3,728 (79.8)	5,239 (60.3)	27,819 (71.1)
Former smoker	3,578 (5.4)	248 (5.3)	1,024 (11.8)	3,615 (9.2)
Current smoker	9,177 (13.8)	697 (14.9)	2,428 (27.9)	7,721 (19.7)
Alcohol consumption				
Never	56,611 (85.2)	4,110 (87.9)	5,740 (66.1)	32,454 (82.9)
Former	1,255 (1.9)	82 (1.8)	252 (2.9)	1,054 (2.7)
Current	8,530 (12.9)	481 (10.3)	2,699 (31.0)	5,647 (14.4)
Excessive^1^	1,520 (2.3)	0 (0.0)	1,619 (18.6)	0 (0.0)
Habit of regular exercise	22,632 (34.1)	1,463 (31.3)	2,981 (34.3)	13,621 (34.8)
Body mass index (kg/m^2^)	21.5±2.6	21.5±1.2	26.4±3.2	26.5±3.4
Overweight	11,241 (16.9)	0 (0.0)	2,406 (27.7)	10,822 (27.6)
Obesity	5,850 (8.8)	0 (0.0)	5,506 (63.4)	24,734 (63.2)
Waist circumference (cm)				
Male	78.2±6.9	77.7±4.5	88.8±7.7	88.8±7.9
Female	68.5±5.9	70.3±4.1	80.4±7.9	80.6±8.1
Prediabetes	19,030 (28.7)	1,346 (28.8)	4,848 (55.8)	21,490 (54.9)
Diabetes mellitus	1,050 (1.6)	0 (0.0)	1,004 (11.6)	4,437 (11.3)
Hypertension	5,103 (7.7)	156 (3.3)	2,405 (27.7)	10,626 (27.1)
Dyslipidemia	9,677 (14.6)	785 (16.8)	3,757 (43.2)	17,316 (44.2)
Estimated glomerular filtration rate (mL/min/1.73 m^2^)	78.7±17.5	78.9±11.4	74.6±11.3	75.2±12.1
Chronic kidney disease	2,890 (4.4)	151 (3.2)	699 (8.0)	3,220 (8.2)
Chronic hepatitis B	6,426 (9.7)	0 (0.0)	4,969 (57.2)	0 (0.0)
Chronic hepatitis C	139 (0.2)	0 (0.0)	104 (1.2)	0 (0.0)
Alanine aminotransferase (U/L)	21.3±19.4	24.0±15.6	43.7±40.0	37.6±27.3
Glutamyl transferase (U/L)	20.5±27.7	23.0±19.2	47.5±76.2	37.1±42.9
Triglyceride (mg/dL)	83.4±49.6	94.7±43.4	160.8±150.3	158.0±117.7
Cholesterol (mg/dL)	188.7±33.0	196.3±33.6	202.0±36.5	203.6±36.2
High-density lipoprotein (mg/dL)	64.1±14.8	60.3±13.1	51.8±11.9	51.6±11.1
Low-density lipoprotein (mg/dL)	110.0±30.2	119.9±31.1	126.1±33.4	128.6±33.6
Fibrosis by Fib-4				
1.30≤Fib-4<2.67	-	492 (10.5)	1,667 (19.2)	5,466 (14.0)
Fib-4≥2.67	-	16 (0.3)	166 (1.9)	306 (0.8)

Values are presented as mean±standard deviation or number (%). Fib-4, fibrosis-4 score; FLD, fatty liver disease; MAFLD, metabolic dysfunction-associated fatty liver disease; NAFLD, non-alcoholic fatty liver disease.

1 Excessive alcohol consumption was defined as more than 70 g to 140 g of alcohol per week; These participants were categorized as current alcohol drinkers.

**Table 2.** Association of all-cause mortality rates with various fatty liver classifications and Fib-4 status

Variables	Participants		Follow-up		Rate^1^
	Event (n)	Total (n)	Total (n)	Mean±SD (yr)	
Overall	2,037	118,915	1,141,694	9.6±1.2	17.8 (17.1, 18.6)
NAFLD/MAFLD status					
Non-FLDs	931	66,396	636,777	9.6±1.1	14.6 (13.7, 15.6)
NAFLD only	45	4,673	45,106	9.7±1.1	10.0 (7.3, 13.3)
Both FLDs	834	39,155	375,774	9.6±1.2	22.2 (20.7, 23.8)
MAFLD only	227	8,691	84,037	9.7±1.2	27.0 (23.6, 38.8)
By NAFLD and Fib-4 status					
No NAFLD	1,158	75,087	720,814	9.6±1.1	16.1 (16.1, 17.0)
NAFLD	879	43,828	420,880	9.6±1.2	20.9 (19.5, 22.3)
Fib-4<1.30	447	37,548	361,088	9.6±1.1	12.4 (11.3, 13.6)
1.30≤Fib-4<2.67	367	5,958	56,978	9.6±1.4	64.4 (58.0, 17.3)
Fib-4≥2.67	76	322	2,814	8.7±2.4	270.0 (212.8, 338.0)
By MAFLD and Fib-4 status					
No MAFLD	976	71,069	681,883	9.6±1.1	14.3 (13.4, 15.2)
MAFLD	1,061	47,846	459,811	9.6±1.2	23.1 (21.7, 24.5)
Fib-4<1.30	523	40,241	387,390	9.6±1.1	13.5 (12.4, 14.7)
1.30≤Fib-4<2.67	440	7,133	68,180	9.6±1.4	64.5 (58.6, 70.9)
Fib-4≥2.67	98	472	4,243	9.0±2.1	231.0 (187.5, 281.5)

Fib-4, fibrosis-4 score; FLD, fatty liver disease; MAFLD, metabolic-dysfunction associated fatty liver disease; NAFLD, non-alcoholic fatty liver disease; SD, standard deviation.

1 Rate (95% confidence interval) per 10,000 person-years.

**Table 3.** Hazard rations for all-cause mortality in participants with FLD by classification and Fib-4 status

Variables	Cox regression models^1^		
	Unadjusted	Model 1	Model 2
FLD by NAFLD/MAFLD definitions			
Both FLDs	Reference	Reference	Reference
NAFLD only	0.45 (0.33, 0.61)	0.84 (0.62, 1.14)	1.08 (0.78, 1.48)
MAFLD only	1.21 (1.04, 1.40)	1.29 (1.11, 1.49)	1.26 (1.09, 1.47)
NAFLD by Fib-4 status			
Fib-4<1.30	Reference	Reference	Reference
1.30≤Fib-4<2.67	5.04 (4.38, 5.79)	0.94 (0.79, 1.13)	0.97 (0.81, 1.16)
Fib-4≥2.67	22.42 (17.58, 28.59)	1.99 (1.48, 2.67)	1.93 (1.44, 2.58)
MAFLD by Fib-4 status			
Fib-4<1.30	Reference	Reference	Reference
1.30≤Fib-4<2.67	4.77 (4.21, 5.42)	1.05 (0.90, 1.24)	1.07 (0.91, 1.26)
Fib-4≥2.67	17.45 (14.06, 21.65)	2.14 (1.65, 2.76)	2.08 (1.61, 2.70)

Values are presented as hazard ratio (95% confidence interval). FLD, fatty liver disease; Fib-4, fibrosis-4 score; MAFLD, metabolic dysfunction-associated fatty liver disease; NAFLD, nonalcoholic fatty liver disease.

1 Model 1: Adjusted for age, sex, marriage, education, and annual household income; Model 2: Additionally adjusted for body mass index, smoking, alcohol drinking, regular exercise, hypertension, dyslipidemia, and chronic kidney disease.

## Supplementary materials

Supplementary Material 1. Cause-specific numbers of death and mortality rates in people with various fatty liver classifications and Fib-4 status

Supplementary Material 2. Flow chart of identifying people with non-alcoholic fatty liver disease (NAFLD) and without NAFLD

Supplementary Material 3. Flow chart of identifying people with metabolic dysfunction-associated fatty liver disease (MAFLD)

Supplementary Material 4. Flow chart of identifying groups of people by combination of non-alcoholic fatty liver disease (NAFLD) and metabolic dysfunction-associated fatty liver disease (MAFLD) definitions

Supplementary Material 5. Comparisons of cumulative all-cause mortality rates in participants (A) with NAFLD by different fibrosis status; (B) with MAFLD by different fibrosis status. Fib-4: fibrosis-4 score; MAFLD: metabolic-dysfunction associated fatty liver disease; NAFLD: non-alcoholic fatty liver disease

Supplementary Material 6. Comparisons of multivariable adjusted hazard ratios of cancer and CVD mortality in participants with NAFLD or MAFLD. BMI: body mass index; CI: confidence interval; Fib-4: fibrosis-4 score; HR: hazard ratio; MAFLD: metabolic-dysfunction associated fatty liver disease; NAFLD: non-alcoholic fatty liver disease
