# Supplementary material for: Comparison of all-cause mortality associated with non-alcoholic fatty liver disease and metabolic dysfunction-associated fatty liver disease in Taiwan MJ cohort
Source: Epidemiol Health. 2024 Jan 21;46:e2024024. doi: 10.4178/epih.e2024024 (PMC11099596; doi:10.4178/epih.e2024024)
Supplement: Supplementary Material 1. — Cause-specific numbers of death and mortality rates in people with various fatty liver classifications and Fib-4 status [file epih-46-e2024024-Supplementary-1.docx]

**Supplementary Material 1.** Cause-specific numbers of death and mortality rates in people with various fatty liver classifications and Fib-4 status

|  | No. of participants and years of follow-up | | | All-cause mortality | | Cancer mortality | | Cardiovascular mortality | | Trauma and self-harm Mortality | | Liver disease related mortality | |
| --- | --- | --- | --- | --- | --- | --- | --- | --- | --- | --- | --- | --- | --- |
|  | No. of participants | Total | Mean ± SD | Event | Rate^1^ | Event | Rate^1^ | Event | Rate^1^ | Event | Rate^1^ | Event | Rate^1^ |
| Overall | 118,915 | 1,141,694 | 9.6 ± 1.2 | 2,037 | 17.8 (17.1-18.6) | 910 | 8.0  (7.5-8.5) | 317 | 2.8  (2.5-3.1) | 174 | 1.5  (1.3-1.8) | 19 | 0.2  (0.1-0.3) |
| By NAFLD/MAFLD status |  |  |  |  |  |  |  |  |  |  |  |  |  |
| Non-FLDs | 66,396 | 636,777 | 9.6 ± 1.1 | 931 | 14.6 (13.7-15.6) | 421 | 6.6  (6.0-7.3) | 123 | 1.9  (1.6-2.3) | 101 | 1.6  (1.3-1.9) | 9 | 0.1  (0.1-0.3) |
| NAFLD only | 4,673 | 45,106 | 9.7 ± 1.1 | 45 | 10.0 (7.3-13.3) | 24 | 5.3  (3.4-7.9) | 7 | 1.6  (0.6-3.2) | 7 | 1.6  (0.6-3.2) | 0 | 0 |
| Both FLDs | 39,155 | 375,774 | 9.6 ± 1.2 | 834 | 22.2 (20.7-23.8) | 353 | 9.4 (8.4-10.4) | 147 | 3.9  (3.3-4.6) | 47 | 1.3  (0.9-1.7) | 6 | 0.2  (0.1-0.3) |
| MAFLD only | 8,691 | 84,037 | 9.7 ± 1.2 | 227 | 27.0 (23.6-30.8) | 112 | 13.3 (11.0-16.0) | 40 | 4.8  (3.4-6.5) | 19 | 2.3  (1.4-3.5) | 4 | 0.5  (0.1-1.2) |
| By NAFLD and Fib-4 status |  |  |  |  |  |  |  |  |  |  |  |  |  |
| Non-NAFLD | 75,087 | 720,814 | 9.6 ± 1.1 | 1,158 | 16.1 (15.2-17.0) | 533 | 7.4  (6.8-8.0) | 163 | 2.3  (1.9-2.6) | 120 | 1.7  (1.4-2.0) | 13 | 0.2  (0.1-0.3) |
| NAFLD | 43,828 | 420,880 | 9.6 ± 1.2 | 879 | 20.9 (19.5-22.3) | 377 | 9.0  (8.1-9.9) | 154 | 3.7  (3.1-4.3) | 54 | 1.3  (1.0-1.7) | 6 | 0.1  (0.1-0.3) |
| Fib-4 < 1.30 | 37,548 | 361,088 | 9.6 ± 1.1 | 447 | 12.4 (11.3-13.6) | 222 | 6.1  (5.4-7.0) | 68 | 1.9  (1.5-2.4) | 40 | 1.1  (0.8-1.5) | 2 | 0.1  (0.0-0.2) |
| 1.30 ≦ Fib-4 < 2.67 | 5,958 | 56,978 | 9.6 ± 1.4 | 367 | 64.4  (58.0-71.3) | 131 | 23.0 (19.2-27.3) | 71 | 12.5 (9.7-15.7) | 14 | 2.5  (1.3-4.1) | 0 | 0 |
| Fib-4 ≧ 2.67 | 322 | 2,814 | 8.7 ± 2.4 | 76 | 270.1 (212.8-338.0) | 24 | 85.3 (54.6-126.9) | 15 | 53.3 (29.8-87.9) | 0 | 0 | 4 | 14.2 (3.9-36.4) |
| By MAFLD and Fib-4 status |  |  |  |  |  |  |  |  |  |  |  |  |  |
| Non-MAFLD | 71,069 | 681,883 | 9.6 ± 1.1 | 976 | 14.3 (13.4-15.2) | 445 | 6.5  (5.9-7.2) | 130 | 1.9  (1.6-2.3) | 60 | 0.9  (0.7-1.1) | 9 | 0.1  (0.1-0.3) |
| MAFLD | 47,846 | 459,811 | 9.6 ± 1.2 | 1,061 | 23.1 (21.7-24.5) | 465 | 10.1 (9.2-11.1) | 187 | 4.1  (3.5-4.7) | 66 | 1.4  (1.1-1.8) | 10 | 0.2  (0.1-0.4) |
| Fib-4 < 1.30 | 40,241 | 387,390 | 9.6 ± 1.1 | 523 | 13.5 (12.4-14.7) | 254 | 6.6  (5.8-7.4) | 84 | 2.2  (1.7-2.7) | 50 | 1.3  (1.0-1.7) | 3 | 0.1  (0.0-0.2) |
| 1.30 ≦ Fib-4 < 2.67 | 7,133 | 68,180 | 9.6 ± 1.4 | 440 | 64.5 (58.6-70.9) | 178 | 26.1 (22.4-30.2) | 83 | 12.2 (9.7-15.1) | 16 | 2.3  (1.3-3.8) | 1 | 0.1  (0.0-0.8) |
| Fib-4 ≧ 2.67 | 472 | 4,243 | 9.0 ± 2.1 | 98 | 231.0 (187.5-281.5) | 33 | 77.8 (53.5-109.2) | 20 | 47.1 (28.8-72.8) | 0 | 0 | 6 | 14.1 (5.2-30.8) |

Fib-4: fibrosis-4 score; FLD: fatty liver disease; MAFLD: metabolic-dysfunction associated fatty liver disease; NAFLD: nonalcoholic fatty liver disease; SD: standard deviation

^1^ Rate (95% confidence interval) per 10,000 person-year
