# Supplementary material for: Comparison of all-cause mortality associated with non-alcoholic fatty liver disease and metabolic dysfunction-associated fatty liver disease in Taiwan MJ cohort
Source: Epidemiol Health. 2024 Jan 21;46:e2024024. doi: 10.4178/epih.e2024024 (PMC11099596; doi:10.4178/epih.e2024024)
Supplement: Supplementary Material 4. — Flow chart of identifying groups of people by combination of non-alcoholic fatty liver disease (NAFLD) and metabolic dysfunction-associated fatty liver disease (MAFLD) definitions [file epih-46-e2024024-Supplementary-4.docx]

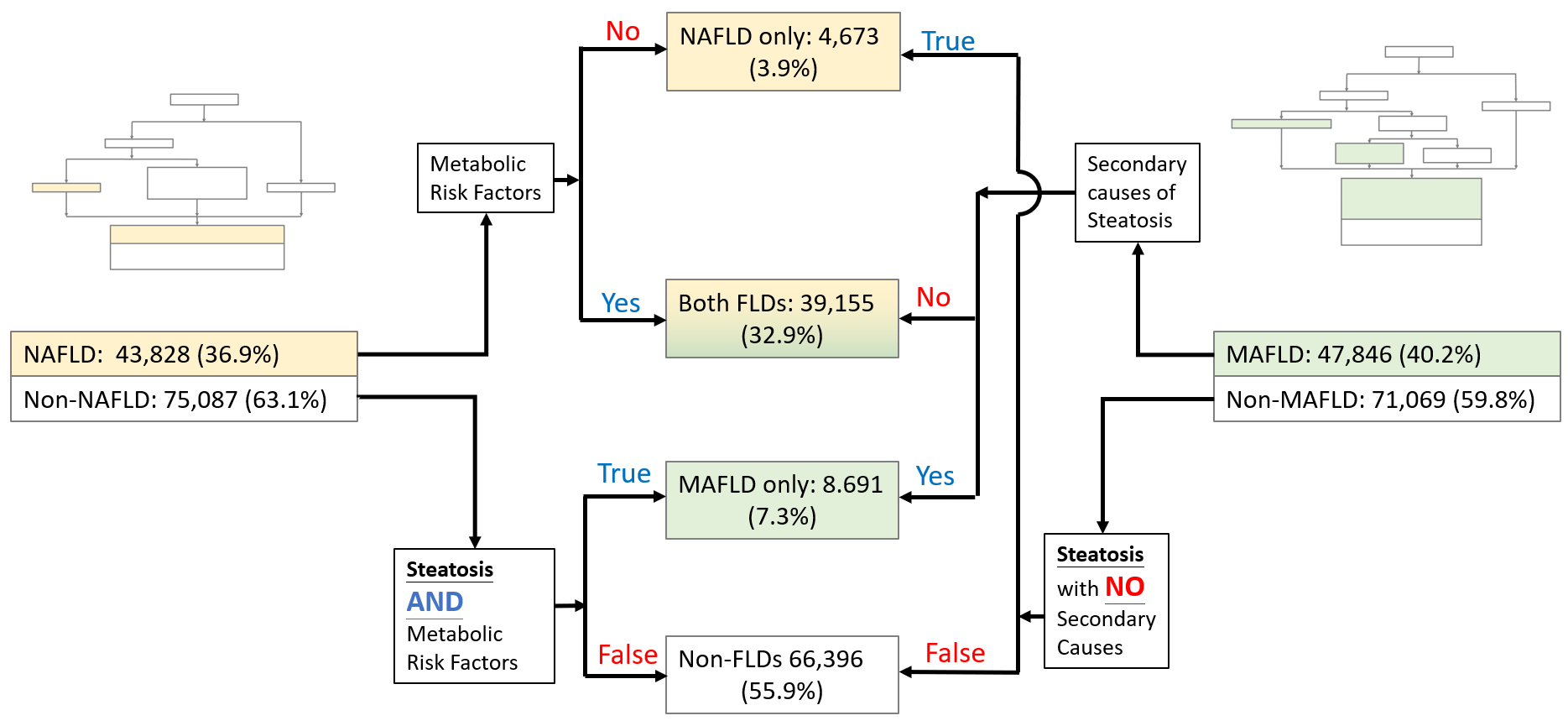


**Supplementary Material 4.** Flow chart of identifying groups of people by combination of non-alcoholic fatty liver disease (NAFLD) and metabolic dysfunction-associated fatty liver disease (MAFLD) definitions
