# Supplementary material for: Comparison of all-cause mortality associated with non-alcoholic fatty liver disease and metabolic dysfunction-associated fatty liver disease in Taiwan MJ cohort
Source: Epidemiol Health. 2024 Jan 21;46:e2024024. doi: 10.4178/epih.e2024024 (PMC11099596; doi:10.4178/epih.e2024024)
Supplement: Supplementary Material 5. — Comparisons of cumulative all-cause mortality rates in participants (A) with NAFLD by different fibrosis status; (B) with MAFLD by different fibrosis status. Fib-4: fibrosis-4 score; MAFLD: metabolic-dysfunction associated fatty liver disease; NAFLD: non-alcoholic fatty liver disease [file epih-46-e2024024-Supplementary-5.docx]

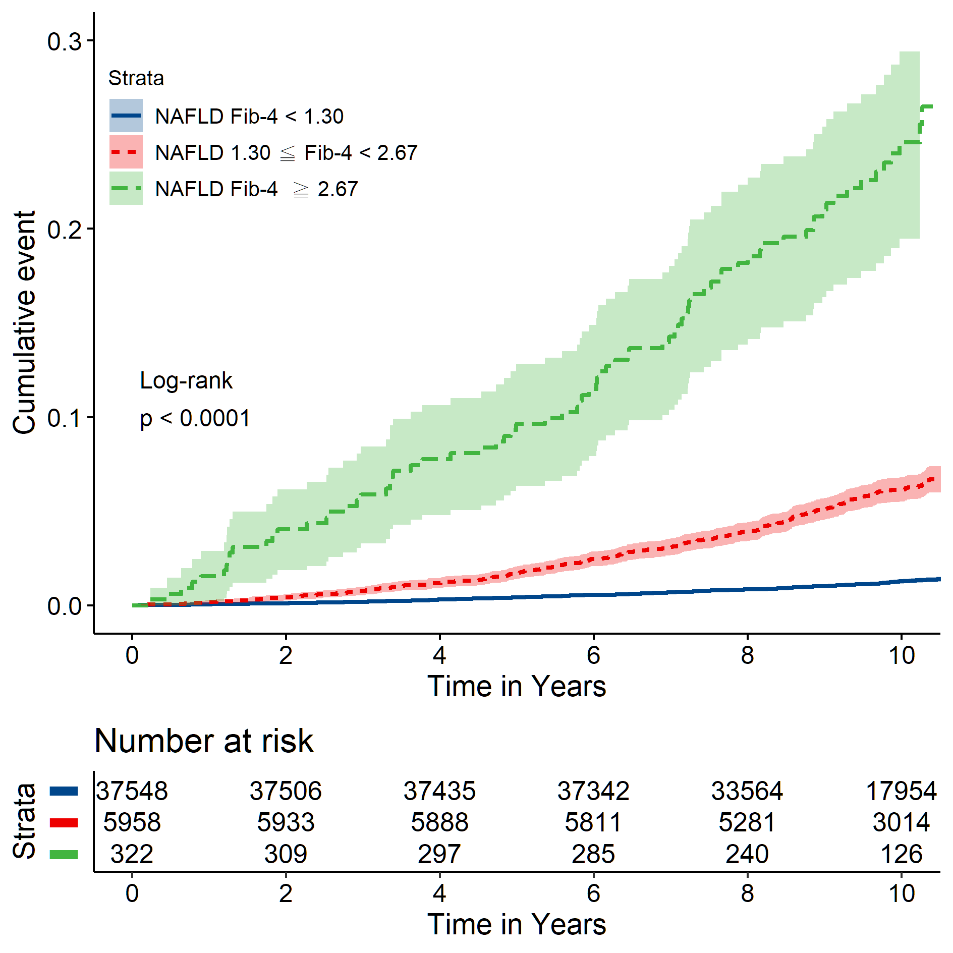

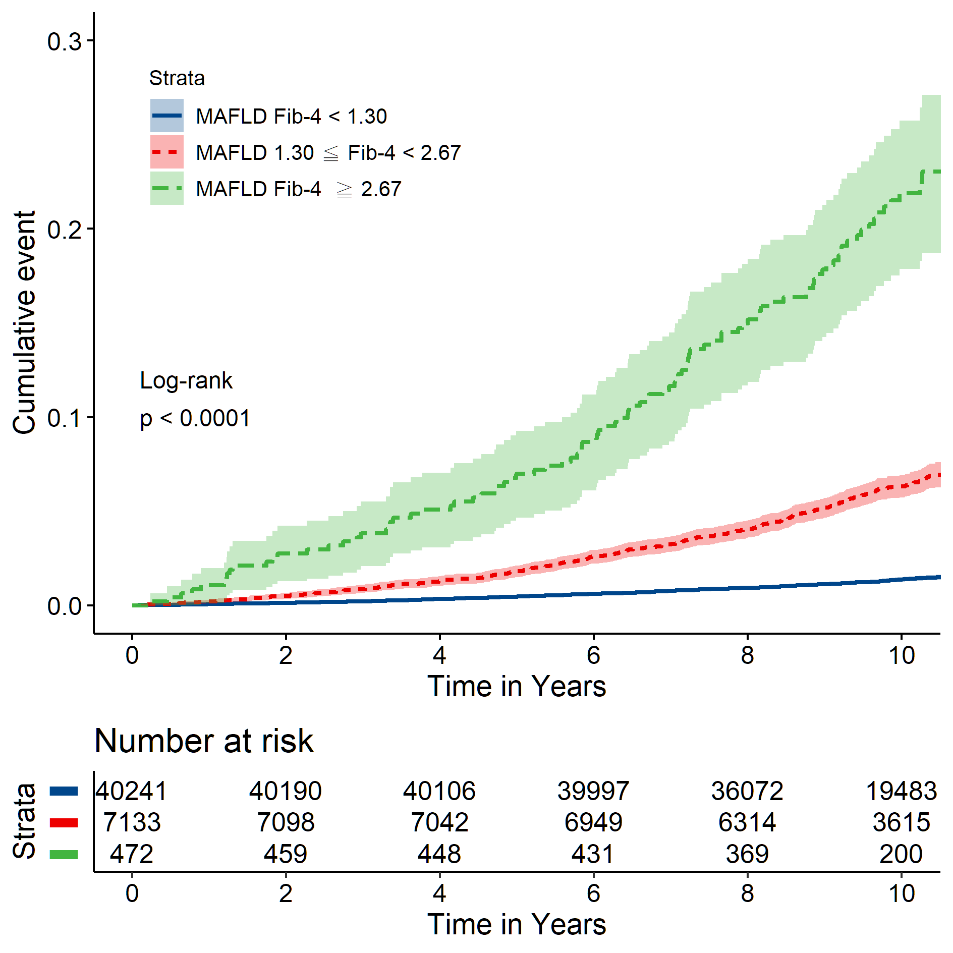


**A B**

**Supplementary Material 5.** Comparisons of cumulative all-cause mortality rates in participants (A) with NAFLD by different fibrosis status; (B) with MAFLD by different fibrosis status.

Fib-4: fibrosis-4 score; MAFLD: metabolic-dysfunction associated fatty liver disease; NAFLD: non-alcoholic fatty liver disease
