# Supplementary material for: Comparison of all-cause mortality associated with non-alcoholic fatty liver disease and metabolic dysfunction-associated fatty liver disease in Taiwan MJ cohort
Source: Epidemiol Health. 2024 Jan 21;46:e2024024. doi: 10.4178/epih.e2024024 (PMC11099596; doi:10.4178/epih.e2024024)
Supplement: Supplementary Material 6. — Comparisons of multivariable adjusted hazard ratios of cancer and CVD mortality in participants with NAFLD or MAFLD. BMI: body mass index; CI: confidence interval; Fib-4: fibrosis-4 score; HR: hazard ratio; MAFLD: metabolic-dysfunction associated fatty liver disease; NAFLD: non-alcoholic fatty liver disease [file epih-46-e2024024-Supplementary-6.docx]

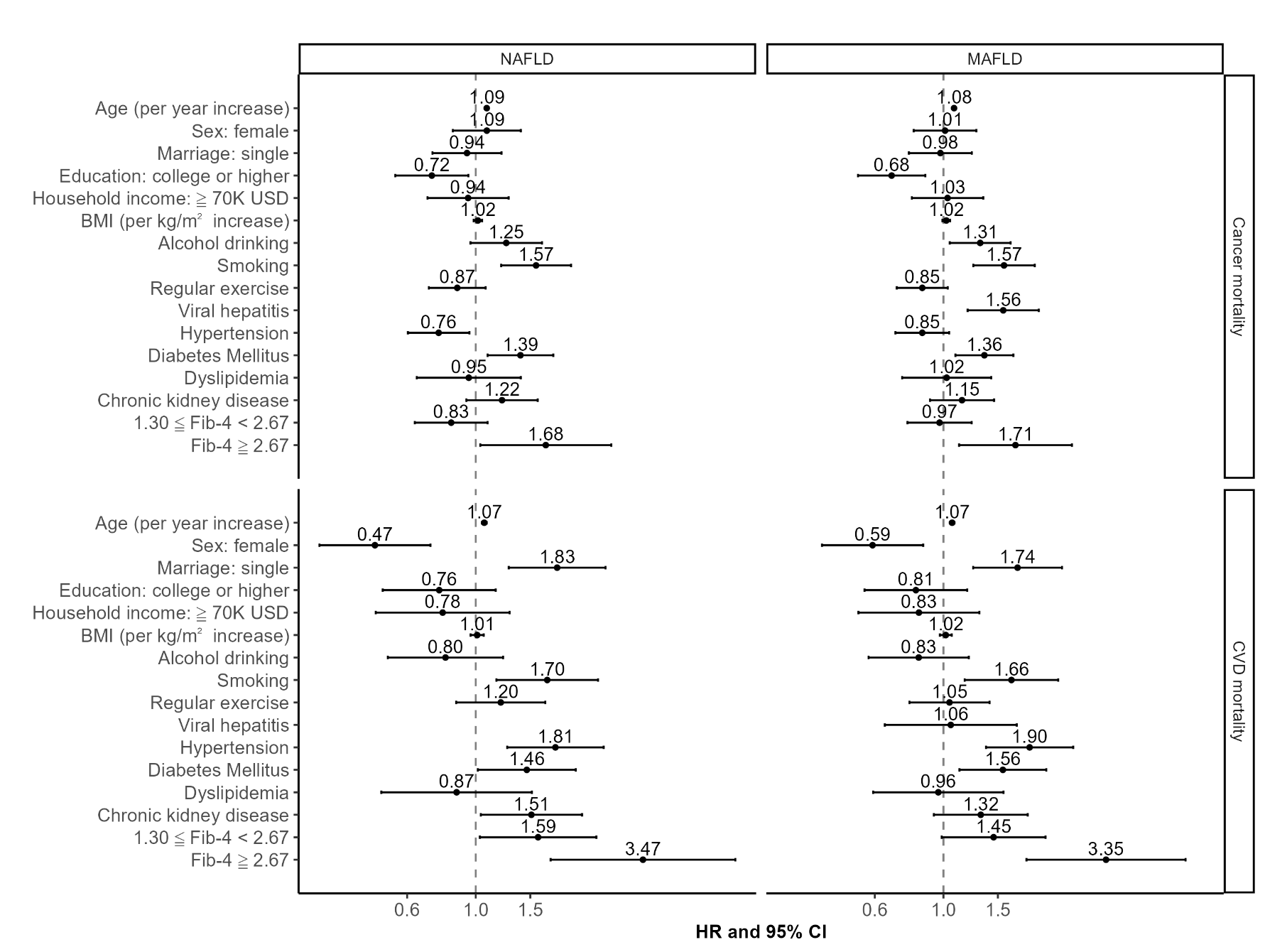


**Supplementary Material 6.** Comparisons of multivariable adjusted hazard ratios of cancer and CVD mortality in participants with NAFLD or MAFLD.

BMI: body mass index; CI: confidence interval; Fib-4: fibrosis-4 score; HR: hazard ratio; MAFLD: metabolic-dysfunction associated fatty liver disease; NAFLD: non-alcoholic fatty liver disease
